# The Reciprocal Relationship between Sleep and Epilepsy

**DOI:** 10.3390/jpm14010118

**Published:** 2024-01-20

**Authors:** Yana Krutoshinskaya, Kelly Coulehan, Galyna Pushchinska, Rebecca Spiegel

**Affiliations:** Department of Neurology, Renaissance School of Medicine at Stony Brook University, Stony Brook, NY 11794, USA; kelly.coulehan@stonybrookmedicine.edu (K.C.); galyna.pushchinska@stonybrookmedicine.edu (G.P.); rebecca.spiegel@stonybrookmedicine.edu (R.S.)

**Keywords:** sleep, epilepsy, anti-seizure medications, obstructive sleep apnea, parasomnia, nocturnal lobe epilepsy, vagus nerve stimulator, ketogenic diet, CPAP, insomnia

## Abstract

The relationship between sleep and epilepsy is bidirectional. Certain epilepsy syndromes predominantly or exclusively manifest during sleep, with seizures frequently originating from non-rapid eye movement (NREM) sleep. Interictal epileptiform discharges observed on electroencephalograms are most likely to be activated during the deep NREM sleep stage known as N3. Conversely, epileptiform discharges, anti-seizure medications (ASMs), as well as other anti-seizure therapies can exert detrimental effects on sleep architecture. Moreover, the co-occurrence of sleep disorders has the potential to exacerbate seizure control. Understating the relationship between sleep and epilepsy is crucial for healthcare providers. Addressing and managing sleep-related problems in individuals with epilepsy can potentially contribute to improved seizure control and overall well-being. At the same time, improving seizure control can improve sleep quality and quantity, thus further improving the health of individuals with epilepsy.

## 1. Introduction

Sleep disturbances frequently coexist with epilepsy, indicating a complex relationship between the two. The relationship between sleep and epilepsy was identified as early as the 4th century BC. Historical records dating back to Hippocrates highlight the cautionary advice given to patients with epilepsy regarding sleep deprivation. Similar advice continues to the present, with numerous contemporary studies demonstrating the provocative nature of sleep deprivation in triggering epileptic events [1]. The bidirectional relationship between sleep and epilepsy results from a variety of shared factors, including changes in neurotransmitter levels, dysregulation of intracellular and extracellular ionic balance, alterations in brain wave frequencies, sleep stage shift alterations, and ineffective feedback loops, to name a few [2,3,4,5].

Given this relationship between sleep and epilepsy, it is important for clinicians treating patients with epilepsy to understand sleep and sleep management for optimal seizure management. Understanding the sleep–epilepsy relationship is also important for clinical characterization. Specifically, sleep-related interictal activity is more suggestive of the seizure onset zone than interictal activity during the awake period. Additionally, although seizures are least likely to occur out of REM sleep, when they do, they can provide the most accurate seizure localization of any sleep stage [6].

It is also important for clinicians to understand how seizure management can impact sleep. Certain anti-seizure medications (ASMs) may possess sedative properties and can influence sleep architecture [7]. Conversely, medications used for the management of sleep disorders, such as sedatives or hypnotics, may interact with ASMs and affect seizure control.

Patients with epilepsy commonly report various sleep-related issues, including poor sleep quality, increase nocturnal awakenings, early morning awakenings, difficulty initiating sleep, and excessive daytime sleepiness. These sleep disturbances can significantly impact the overall quality of life for individuals with epilepsy.

Sleep Architecture: Sleep can be divided into non-rapid eye movement (NREM) sleep and rapid eye movement (REM) sleep. NREM sleep is further divided into stage 1 sleep, stage 2 sleep, and slow-wave sleep (SWS; stage 3). By and large, synchronous brain activity is characteristic of NREM sleep, whereas asynchronous brain activity occurs during REM sleep [8,9]. See Table 1 for the characteristics of the sleep stages [10,11].

Epilepsy Effects on Sleep: A seizure results in an overly abundant unloading of neurochemicals that has the potential to cause neuronal reorganization and alter neuronal functioning. Repeated alterations in neuronal functioning resulting from recurrent seizure activity can lead to alterations of typical sleep functions.

Both diurnal and nocturnal seizures activity affect sleep architecture [12]. Research has consistently shown that there is a reduction in REM sleep and delay in the first REM stage latency following a seizure occurrence [13,14,15]. Seizures are associated with changes in NREM sleep, including increased stage 1 and 2 sleep as well as decreased SWS. Additionally, epileptiform discharges in the absence of clinical seizures can alter the organization of sleep architecture in both NREM and REM sleep.

Sleep Effects on Epilepsy: Typical sleep activity is associated with a decrease in seizure threshold due to the neurological changes that are characteristic of sleep. Specifically, diffuse cortical synchronization of NREM sleep can facilitate interictal epileptiform abnormalities [16]. Meanwhile, this heightened synchronous neuronal firing is beneficial for the generation of the sleep spindles and K-complexes that are characteristic of stage 2. During cycles of NREM and REM sleep, the brain undergoes dramatic alterations in neurochemistry and neuronal functioning. Particularly noteworthy when considering the sleep–epilepsy relationship is the neuronal hypersynchronicity characteristic of NREM sleep and the neuronal hyposyncronicity characteristic of REM sleep. NREM and REM characteristics are outlined in Table 1.

Heightened synchronous neuronal firing, characteristic of NREM sleep, is beneficial for the generation of sleep spindles and K-complexes; however, it leaves the brain predisposed to seizure activity. Hypersynchronization of NREM sleep can lead to hyperexcitability of the cortex, thus facilitating thalamocortical oscillations to transform from sleep spindles into spike waves [17,18], which are characteristic of epileptiform activity. Therefore, not surprisingly, spindle activity and electrochemical changes underlying cerebral synchronization are associated with the spike and wave discharges seen during seizures [17]. In contrast, REM sleep involves an inhibition of thalamocortical synchronizing and therefore an attenuation of synchronous epileptiform discharges [19].

Atypical sleep activity, such as sleep deprivation, can also alter seizure thresholds. Even mild sleep loss can lead to a shortening of latencies from lighter stages of NREM sleep to deeper SWS [5] and decreased time in asynchronous REM sleep. Furthermore, sleep deprivation is associated with increases in seizure frequency and duration.

Effects of Interictal Discharges on Sleep: Interictal epileptiform discharges have the potential to disrupt the intricate signaling mechanisms involved in sleep circuitry. This disruption can lead to sleep fragmentation, perturbing the coordinated physiological processes of sleep. Consequently, there is a proposed hypothesis that these nocturnal interictal discharges may exert an influence on daytime learning, implying a potential impact on cognitive functioning.

Several studies have established associations between nocturnal seizure activity and complaints of disrupted sleep and daytime symptoms. In adults, frequent interictal discharges during sleep are frequently associated with increased daytime sleepiness. Administration of ASMs have been shown to decrease the frequency of nocturnal discharges and ameliorate daytime symptoms.

## 2. Common Epilepsy Syndromes Associated with Sleep

One in five patients with epilepsy has seizures exclusively during sleep. Nineteen percent of generalized seizures occur during sleep, as compared to 51% of localization-related seizures. Temporal lobe epilepsy is the most common sleep-related epilepsy, not because of a particular sleep-related predilection, but because of the common nature of this seizure type. That said, frontal lobe seizures have the greatest penchant to occur out of sleep. Approximately 60% of frontal lobe seizures begin during sleep, as opposed to only 10% of temporal lobe seizures [20].

Many patients with epilepsy experience seizures out of sleep, or “awakening epilepsy”. In fact, up to 90% of seizures occur within 2 h of waking [21]. When seizures occur out of sleep, patients often awaken with a focal aware seizure then progress to a typical focal unaware seizure. Moreover, seizures are more likely to generalize when they originate from sleep.

The transition between light sleep and waking, or vice versa, is often an unstable time due to neurochemical changes related to alterations in arousal mechanisms that can increase susceptibility to epileptiform activity. Seizures and epileptiform abnormalities are typically observed during sleep stage transitions, and unstable sleep is characterized by cortical arousals.

Commonly occurring sleep-related epilepsies include:

- Nocturnal frontal lobe epilepsy
- Nocturnal temporal lobe epilepsy
- Self-limited epilepsy with centrotemporal spikes (SeLECTS; formerly known as benign Rolandic epilepsy or benign epilepsy with centrotemporal spikes)
- Self-limited epilepsy with autonomic seizures (SeLEAS; formerly known as Panayiotopoulos Syndrome)
- Epileptic encephalopathies
- Lennox–Gastaut syndrome
- Landau–Kleffner syndrome
- Developmental/epileptic encephalopathy with spike wave activation in sleep (DEE-SWAS)
- Generalized epilepsies
- Epilepsy with generalized tonic–clonic seizures alone
- Juvenile myoclonic epilepsy

Nocturnal frontal lobe epilepsy (NFLE): NFLE can occur spontaneously or via a familial variant, which is known as autosomal dominant nocturnal frontal lobe epilepsy (ADNFLE). ADNFLE is a distinct clinical syndrome with a typical onset in late childhood that persists throughout life. The disorder demonstrates the autosomal dominant inheritance pattern, with an approximate penetrance of 70%. A total of 10–20% of patients with ADNFLE have mutations in the neuronal nicotinic acetylcholine receptor alpha 4 (CHRNA4) subunit. Two genetic loci that have been identified are 20q13.2-3 and 15q24. These patients appear to respond to sodium channel blocking ASMs such as carbamazepine, oxcarbazepine, lacosimide, topiramate, or lamotrigine [22].

The ADNFLE is characterized by brief stereotyped hyperkinetic or tonic motor seizures that occur in clusters during sleep following sudden arousals. Seizures are brief and usually last less than one minute. Usually, there is a lack of ictal and interictal EEG correlates (apart from movement and muscle artifacts associated with the spells) because seizures involve deep medial frontal generators.

NFLE, in general, can be difficult to differentiate from NREM sleep parasomnias, requiring polysomnography with video monitoring to document the stereotypy of the attacks and abnormal motor aspects of the seizures. NREM sleep parasomnias consist of disorders of arousal, which include sleepwalking, sleep terrors, and confusional arousals. Such parasomnias tend to emerge in early childhood, whereas NFLE typically occurs around the ages of 10–14. Furthermore, NFLE seizures are characterized by frequent nightly occurrences, repetitive episodes throughout the night, and brief events (30 s–2 min). In contrast, sleep terrors are infrequent, isolated, and usually have prolonged durations (≥30 min). Importantly, sleep terrors commonly occur during the transition from SWS to REM sleep.

The motor patterns observed in NFLE can be bizarre but very stereotypical and may include ballistic movements and dystonic posturing, which differentiate them from the partial and sudden arousals seen in parasomnias. Additionally, while parasomnias tend to diminish with age, NFLE persists into adulthood, further distinguishing the two conditions. NFLE usually lacks, or has very brief, post-events confusion, and is frequently characterized by agitation. NREM parasomnia presents with a prolonged confusion state after the event [12,23].

Nocturnal temporal lobe epilepsy: In general, compared to diurnal temporal lobe epilepsy, nocturnal temporal lobe epilepsy is thought to portend a more favorable outcome following epilepsy surgery, and patients exhibit less frequent seizures [24].

**Figure 1:**
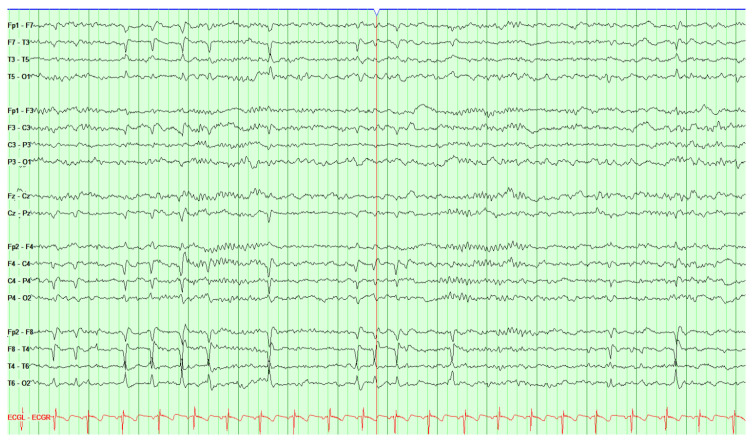
Sleep activation of focal interictal epileptiform activity in a 9-year-old child with benign centrotemporal epilepsy. A train of repetitive focal interictal epileptiform activity in the form of centro-temporal spike discharges during N2 sleep. The example shown is a 15 s screen, with a sensitivity of 15 uv/mm, HFF = 70 Hz, LFF = 1 Hz; longitudinal bipolar montage. The read lead is ECG. The black are EEG leads.

Self-limited epilepsy with centrotemporal spikes SeLECTS: SeLECTS is typically associated with seizures manifesting as focal spasms of the face or hands with jerking, occurring in the first one-third of the night. These events classically start in NREM sleep and, for many patients, the interictal discharge appears only during sleep (Figure 1). Seizure onset typically occurs at approximately 6 to 8 years old, and seizures commonly resolve in adolescence.

Self-limited epilepsy with autonomic seizures (SeLEAS): SeLEAS is a childhood epilepsy syndrome with seizure onset usually between 3 and 6 years of age. It is characterized by focal, mainly autonomic seizures. The autonomic seizures consist of pallor, nausea, retching, and vomiting with or without impaired awareness. Sympathetic responses predominate during most seizures, causing tachycardia, tachypnea, increased blood pressure, pupillary dilatation, diaphoresis, and facial flushing [25]. Rarely, visual symptoms, such as hallucinations or loss of vision, may be presented during the seizures [26].

However, ictal parasympathetic activity or sympathetic inhibition can predominate, causing increased salivation, gastric acid secretion, peristalsis, miosis, reduced heart and respiratory rates, and decreased blood pressure. Combinations of sympathetic and parasympathetic activation and inhibition may occur simultaneously or sequentially during seizures. Most seizures are nocturnal. Electroencephalography reveals interictal multifocal stereotyped spikes and spike and waves discharges with posterior accentuation that are amplified by sleep. Half of the seizures can last for more than 60 min, often 2 to 3 h, and thus constitute autonomic status epilepticus. 

SeLEAS is a remarkably benign condition despite the high incidence of autonomic status epilepticus. Seizures are self-limiting, with remission typically occurring within a few years from onset, but approximately 10% of patients may have more protracted active seizure periods [27,28,29].

Epileptic encephalopathies: Epileptic encephalopathies are severe seizure disorders associated with progressive cerebral dysfunction resulting in functional decline. There are eight recognized epileptic encephalopathies, three of which are closely related to sleep.

Lennox–Gaustaut Syndrome is characterized by mixed seizures with primarily nocturnal tonic seizures, diffuse slow spike and wave discharges, and intellectual disability. The prototypical seizure is a nocturnal generalized tonic, but atypical absence is also very common. Drop attacks or astatic seizures are another common feature [30]. The age of onset is early childhood, typically between the ages of 3 and 5 years.

Landau–Kleffner syndrome (LKS): LKS is a rare epilepsy syndrome characterized by language regression and an abnormal EEG [31]. In the classic presentation, LKS usually develops between the ages of 2 and 8 years and is associated with acquired aphasia. Seizures and behavioral disturbance, particularly hyperactivity, are common features. EEG in LKS shows bilateral, multifocal spikes and spike and wave discharges, usually occurring in the posterior regions, especially the temporal regions, with marked activations in N3 sleep [32]. Ictal epileptiform discharges may occur in many locations and may even be generalized. The characteristic language deficit might develop as a result of abnormal metabolism from the enduring epileptiform discharges [33].

Developmental and/or epileptic encephalopathy with spike wave activation in sleep (DEE-SWAS, EE-SAWS): This type of epilepsy was previously known as continuous spike-waves discharges during sleep (CSWS), electrical status epilepticus in sleep (ESES), or epilepsy with continuous spike-waves during slow-wave sleep [34]. DEE-SWAS is characterized by different types of seizures, neurocognitive regression, behavioral issues, and an EEG pattern of electrical status epilepticus during sleep (ESES). ESES is characterized by marked sleep potentiation of epileptiform activity in the transition from wakefulness to sleep that leads to near-continuous bilateral (or occasionally lateralized) slow spikes and waves that occupy a significant proportion of nonrapid eye movement (non-REM) sleep [35,36].

The exact reason for the sleep activation of EEG abnormalities is unknown. Some researchers have correlated abnormal EEG signatures with abnormal hyperactivation of the thalamic oscillatory circuit: an interplay between inhibitory GABAergic reticular thalamic neurons and excitatory glutaminergic dorsal thalamic neurons. Researchers have also hypothesized that the change in the thalamic oscillatory circuit might be due to a substitution from the GABA-A to GABA-B-mediated post-synaptic inhibition. Sleep potentiation of epileptiform discharges may disrupt cortical information processing and trigger learning and memory impairments [37].

DEE-SWAS is a rare form of epilepsy. It starts in children between 2 and 12 years of age, with a peak between 3 and 5 years, and the course of disease is variable. ASMs often control seizures, but language, cognitive, and behavior issues are more difficult to treat. EEG activity usually improves during adolescence and may be accompanied by some cognitive and behavioral improvement.

The subgroup of ESES patients with structural brain pathology such as perinatal infarction or cortical malformation may benefit from epilepsy surgery. Different surgical procedures, including multiple subpial transections, focal lesionectomies, and hemispherectomies, have been utilized mainly in patients with structural brain pathologies. Children with unilateral brain pathologies were frequently seizure-free after receiving a hemispherectomy or focal resection, with the disappearance of the EEG pattern and improvement in cognitive status [38].

**Figure 2:**
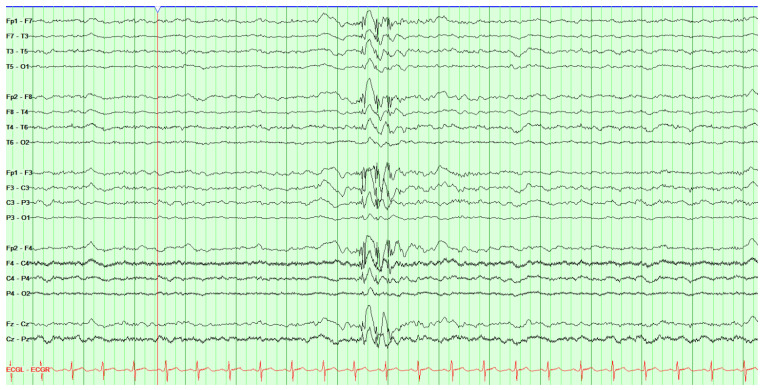
Electroencephalogram showing activation of generalized epileptiform activity. Generalized spike and wave discharges typical of juvenile myoclonic epilepsy (JME). Example shown is a 15 s screen, with a sensitivity of 10 uv/mm, HFF = 70 Hz, LFF = 1 Hz; bipolar montage.

Juvenile myoclonic epilepsy (JME) is characterized by either myoclonic or bilateral tonic–clonic seizures that most commonly occur shortly after awakening. The EEG pattern includes generalized atypical spike-wave discharges (Figure 2). It is not uncommon that subtle myoclonus, especially in the morning, is mistaken for simple clumsiness for many years before the true diagnosis is recognized [39].

## 3. Comorbid Sleep Disorders in Epilepsy

Epilepsy exerts notable effects on sleep, influencing sleep architecture and overall sleep quality. Seizures elicit postictal somnolence. Moreover, seizures can disrupt sleep continuity by inducing increased wakefulness after sleep onset, sleep fragmentation, and suppression of REM sleep after the seizure. These effects are not limited to the immediate postictal phase but can extend to interictal periods as well. Compared to the general population, individuals with epilepsy experience a considerably higher prevalence of sleep disturbances [40].

These disturbances manifest as increased sleep latency and an elevated frequency of nocturnal awakenings. Furthermore, epileptic seizures, interictal epileptiform discharges, and adverse effects of ASMs can induce alterations in normal sleep architecture.

Insomnia can also affect patients with epilepsy. Arousals can be from multiple factors, including epilepsy itself, ASMs effects, or other substances. Patients can also have fears associated with sleep, such as having a seizure out of sleep [41]. Frequent arousals have been shown to be a trigger and manifestation of seizures themselves [42].

Patients with epilepsy exhibit a significantly higher incidence of anxiety and depression when compared to the general population. These psychiatric comorbidities are frequently associated with the presence of insomnia in individuals with epilepsy. The interplay between epilepsy, anxiety, depression, and insomnia underscores the complex relationship between neurological and psychological factors in this patient population.

Excessive daytime sleepiness is a common complaint amongst epilepsy patients. Sleep-disordered breathing, such as obstructive sleep apnea, is thought to contribute to excessive daytime sleepiness in some epilepsy patients [43].

Obstructive sleep apnea. Polysomnographic examinations conducted on individuals with epilepsy have revealed a high prevalence of OSA. OSA is a disorder characterized by the recurrent collapse of the upper airway during sleep, often accompanied by sleep fragmentation. Left untreated, OSA can result in significant morbidity [44].

The diagnosis and treatment of comorbid OSA present an opportunity to improve seizure frequency and overall quality of life in patients with epilepsy [45].

It is believed that the presence of OSA itself may contribute to the exacerbation of epilepsy. The potential mechanisms underlying this relationship involve alterations in sleep architecture, sleep deprivation, and hypoxia. Furthermore, seizure frequency is positively associated with the length of apnea events, regardless of the specific epilepsy syndrome [46].

Consequently, a vicious cycle can be observed, wherein epilepsy may increase the likelihood of developing OSA, and OSA, in turn, may worsen epilepsy symptoms.

Encouragingly, it is possible to break this cycle. In adults, the treatment of OSA using positive airway pressure (PAP) therapy has been reported to lead to a reduction in seizure frequency [47]. This suggests that effectively managing OSA in individuals with epilepsy can have a positive impact on their condition.

Several case series and one randomized pilot trial have revealed that addressing comorbid sleep problems was associated with improvements in intractable epilepsy [44,48,49,50]. Treatment of OSA in individuals with epilepsy, typically through the use of PAP therapy such as continuous positive airway pressure (CPAP), can result in improvements in excessive daytime sleepiness, even in the absence of changes or higher doses of ASMs. Excessive daytime sleepiness is a common symptom experienced by patients with both epilepsy and OSA.

The exact mechanisms by which OSA treatment improves excessive daytime sleepiness in individuals with epilepsy are not yet fully understood. However, it is believed that the restoration of normal breathing patterns and alleviation of sleep fragmentation that result from successful OSA treatment contribute to the reduction in daytime sleepiness. Another study revealed that children with epilepsy who were treated surgically for their obstructive sleep apnea demonstrated a 53% median seizure reduction, with approximately one-third becoming seizure-free [51].

## 4. Effect of Antiseizure Medication (ASM) on Sleep

Many medications used to treat epilepsy influence sleep, yet most need further study in both patients with epilepsy and in normal controls. Despite ongoing difficulties in interpreting the evidence, it appears that certain ASMs might yield beneficial outcomes for sleep and could be correlated with achieving seizure control. Nevertheless, in certain scenarios, some ASMs may be connected to deteriorated sleep, while many are linked to the occurrence of insomnia and/or daytime sleepiness. Clinicians should take these factors into account when deciding on treatments, especially for patients with pre-existing sleep disorders (See Table 2) [52]. Medications such as phenobarbital, phenytoin, valproate, and carbamazepine have soporific effects, but they also may produce significant changes in overall sleep architecture, such as decreased REM sleep or increase sleep fragmentation. Pregabalin and gabapentin both appear to have a benefit by increasing SWS, and they may improve sleep and attention in patients with epilepsy and insomnia. But, at the same time, the use of gabapentin, pregabalin, valproic acid, and vigabatrin is associated with weight gain, and this can independently increase the risk of conditions such as OSA [44,52].

Benzodiazepines, particularly diazepam and clobazam, are some of the most commonly used ASMs for ESES. A group of researchers treated 29 children with ESES with oral high-dose diazepam (1 mg/kg, maximum of 40 mg) and noted a reduction in the spike-wave index from 76.7% to 40.8% within 24 h. These patients received 0.5 mg/kg of diazepam for three weeks after the initial high dose before gradual tapering [53]. The other common ASMs used for the treatment of ESES include valproate, ethosuximide, and levetiracetam. ASMs such as phenytoin, phenobarbital, carbamazepine, and oxcarbazepine can worsen seizure control in ESES and require withdrawal in the presence of a worsening drop or absence of seizures.

Felbamate, Lamotrigine, and Zonisamide at high doses may cause insomnia. At lower doses, however, Lamotrigine and Zonisamide appear to have little overall effect on sleep [52].

Preliminary evidence suggests that adjusting medication dosages based on chronopharmacological principles, particularly with a focus on prioritizing sedating medications at night, may offer a promising avenue for improving seizure responses and reducing side effects in individuals with epilepsy [54]. Continued research in this area will provide a more comprehensive understanding of the potential benefits and optimal strategies for implementing chronopharmacology in epilepsy management.

## 5. Ketogenic Diet

The ketogenic diet is a therapeutic approach that has been employed as a strategy to improve seizure control in certain cases of refractory epilepsy. While its primary focus is reducing seizure frequency and severity, there is some evidence to suggest that it may have effects on sleep as well [55].

Although research on the specific effects of the ketogenic diet on sleep independent of improved seizure frequency is limited, some studies have reported improvements in sleep quality and alterations in sleep architecture in individuals following this dietary intervention. These improvements in sleep parameters may be associated with the overall reduction in seizure activity observed in individuals on the ketogenic diet [56].

The mechanisms underlying the potential impact of the ketogenic diet on sleep are not yet fully understood. It has been proposed that the diet’s alteration of metabolism, including the production of ketone bodies, may influence neurotransmitter balance and neuronal excitability, which could indirectly affect sleep regulation [57].

However, it is important to note that the available studies exploring the effects of the ketogenic diet on sleep are limited in scope and often conducted within the context of epilepsy management. Further research is needed to specifically investigate the direct effects of the ketogenic diet on sleep independent of seizure control.

## 6. Vagus Nerve Stimulation

Vagus nerve stimulation (VNS) is a therapeutic approach used for the treatment of certain refractory epilepsies. While its primary aim is to improve seizure control, there is evidence suggesting that VNS may also have effects on sleep parameters.

Several studies have reported that VNS can increase the percentage of slow-wave sleep. Additionally, these studies have observed reductions in daytime sleepiness in individuals undergoing VNS therapy. However, it is important to note that these studies did not specifically analyze the effects of VNS on sleep independent of improved seizure frequency [58,59].

It is worth mentioning that VNS therapy can also have potential implications for sleep-related breathing disorders. VNS has been reported to potentially increase airway disturbances during sleep in some patients by increasing airway resistance due to increased lateral laryngeal muscle tone or by interfering with respiratory sensory feedback [60,61,62].

VNS may increase the risk of developing conditions such as obstructive sleep apnea, central sleep apnea, hypoventilation, and shallow breathing. These effects on respiratory function during sleep can have significant implications for overall sleep quality and daytime functioning [63].

Further research is needed to comprehensively analyze the effects of VNS on sleep independent of seizure control and to better understand the underlying mechanisms involved.

## 7. Conclusions

Sleep in the epileptic brain is inherently unstable. This instability promotes seizures, and seizures in turn fragment sleep, thus facilitating the epileptic process. As such, sleep disturbances frequently coexist with epilepsy and can hinder the management of seizures, while epilepsy can disturb regular sleep patterns, leading to the initiation and exacerbations of sleep disorders and alterations in sleep architecture.

Most sleep-related seizures and interictal epileptiform activity occur out of NREM sleep, with NREM sleep stage N2 being the most common, while rapid eye movement (REM) sleep tends to decrease seizure and interictal epileptiform discharge occurrence.

The use of ASMs and other therapeutic modalities such as VNS can both improve and hinder sleep. It is important to further screen patients for insomnia, daytime sleepiness, and respiratory events as new anti-seizure therapies are introduced or therapy is escalated.

Providers should also be mindful that sleep disorders are common comorbidities in individuals with epilepsy, especially OSA. By recognizing the presence of comorbid sleep disorder in patients with epilepsy, implementing appropriate treatment strategies, and promptly referring patients to sleep specialists when appropriate, healthcare providers can potentially improve seizure control, improve quality of life, improve cognitive functioning, and enhance the overall well-being of these individuals with epilepsy. This underscores the importance of addressing both epilepsy and sleep disorders comprehensively to achieve optimal outcomes.

## Figures and Tables

**Table 1:** Effects of sleep disruption and epileptiform activity on sleep stages [10,11].

	Sleep Phase	Predominant Brain Wave Activity	Frequency (Hertz)	Neuronal Activity	Effects of Sleep Disruption on Sleep Phases	Effects of Epileptiform Activity on Sleep Phases
NREM	Stage N1	Alpha waves Theta waves	8–13 4 to less than 8 Hz	Synchronous (↓ acetylcholine)	Hyper-synchronicity; shorter latencies between stages; increased arousals	Increase in stage 1
	Stage N2	Spindles	11–16			Increase in stage 2; Reduced density or abnormal sleep spindles and K-complexes; Decrease number of stage shifts
		K-complexes	High-voltage, biphasic wave with duration of 0.5 s or more			
	Slow-wave sleep (SWS)	Delta waves (range in slow-wave sleep)	0.5–2 Hz			Reduction in SWS; Increase in SWS shifts
		Slow-wave oscillations	<1			
REM		Theta waves Beta waves Gamma waves [11]	4 to less than 8 Hz Greater than 13 Hz Greater than 25 Hz	Asynchronous (↑ acetylcholine)	Decrease time in REM sleep	Fragmented REM; Increased latency to REM; Decrease time in REM sleep

**Table 2:** Effect of antiseizure medications on sleep.

Drug	Sleep Latency	Stage N2	Slow-Wave Sleep	REM	Sleep Efficiency
Phenytoin	Decrease	Increase	Decrease	Decrease	No change
Valproate					Decrease
Phenobarbital	Decrease	Increase	No change	No change or decrease	No change
Carbamazepine	No change	No change	No change	No change	No change
Gabapentin	No change	No change	Increase	Increase	No change
Lamotrigine	No change; insomnia at a high dose	Increase	Decrease	Increase	No change
Zonisamide	No change; insomnia at a high dose	No change	No change	No change	No change
Topiramate	Decrease	No change	No change	No change	No change
Keppra	NR	NR	Increase	NR	NR
Pregabalin	NR	NR	Increase	NR	Increase
Ethosuximide	No change	No change	Decrease	No change	No change

REM = Rapid eye movement; NR = not reported; Ref. [52].

## Data Availability

No new data were created or analyzed in this study. Data sharing is not applicable to this article.
